# Association of *GSTP1* Ile105Val Polymorphism and Risk of Head and Neck Cancers: A Meta-Analysis of 28 Case-Control Studies

**DOI:** 10.1371/journal.pone.0048132

**Published:** 2012-11-07

**Authors:** Juntian Lang, Xicheng Song, Jinwei Cheng, Shuwei Zhao, Jingping Fan

**Affiliations:** 1 Department of Otorhinolaryngology-Head & Neck Surgery, Shanghai Changzheng Hospital, Second Military Medical University, Shanghai, China; 2 Department of Otolaryngology-Head and Neck Surgery, Yuhuangding Hospital of Qingdao University, Yantai, China; 3 Department of Ophthalmology, Shanghai Changzheng Hospital, Second Military Medical University, Shanghai, China; Harvard Medical School, United States of America

## Abstract

**Background and Aims:**

The Glutathione S*-*transferase P1 (*GSTP1*) polymorphism have been considered a risk modifier for developing head and neck cancer (HNC) in many studies; however, the results of such studies are inconsistent. The aim of this study was to evaluate the possible association between the *GSTP1* Ile105Val polymorphism and risk of HNC.

**Method:**

We performed a search in the relevant electronic database and a meta-analysis based on 28 published case–control studies that included 6,404 cases and 6,523 controls. To take into account the possibility of heterogeneity across the studies, a Chi-square based I^2^-statistic test was performed. Crude pooled odds ratios (ORs) with 95% confidence intervals (CIs) were assessed using both fixed-effects and random-effects models.

**Results:**

The results of this meta-analysis showed that the *GSTP1* Ile105Val polymorphism was not significantly associated with risk of HNC in the overall study population (pooled OR 1.00, 95% CI 0.92–1.09) or in subgroup analyses stratified by ethnicity, sample size, tumor site or publication year. Moreover, substantial evidence of heterogeneity among the studies was observed. Publication year was identified as the main cause of heterogeneity.

**Conclusion:**

This meta-analysis does not support a significant association between the *GSTP1* Ile105Val polymorphism and risk of HNC.

## Introduction

Head and neck cancer (HNC), including cancers of the oral cavity, pharynx, and larynx, is the sixth most common cancer worldwide, with an annual incidence of 500,000 cases [1]. The age-standardized incidence rates in developed and developing countries are 28.4 and 20.6 per 100,000 population, respectively [2]. The development of HNC is a multifactorial process associated with a variety of risk factors. Exposure to tobacco smoke and the consumption of alcohol are considered to be the most important etiological factors in the development of HNC [3]–[5]. However, not every smoker and/or alcohol consumer develops HNC, which suggests that genetic host factors might also contribute to its carcinogenesis.

Recent evidence indicates that carcinogen-metabolizing genes and DNA-repair genes play critical roles in determining individual susceptibility to HNC. Polymorphisms in such genes that encode enzymes may increase or decrease carcinogen activation/detoxification and modulate DNA repair capacity, possibly by altering their expression and function. One of the most important systems in detoxification is the glutathione S-transferase (GST) family of enzymes. *GSTs* are phase II xenobiotic metabolizing enzymes involved in catalyzing the conjugation reactions of reactive intermediates of electrophilic compounds with cytosolic glutathione (GSH). Based on sequence similarities, human cytosolic *GSTs* are mainly coded for at 5 loci: *GSTA* (a), *GSTT1* (h), *GSTM1* (l), *GSTP1* (p), and *GSTM3* (c). *GSTP1* is a major *GST* isoform that catalyzes the conjugation of glutathione to toxic compounds, resulting in more water-soluble and less biologically active products that are easily excreted.

*GSTP1* is located on chromosome 11q13. To date, three polymorphic alleles of *GSTP1* are known–GSTP1*B, GSTP1*C, and GSTP1*D–in addition to the wild-type allele, GSTP1*A [6]. GSTP1*B alleles have an A-to-G transition at nucleotide 313 (codon 105, exon 5), causing an isoleucine-to-valine change, while GSTP1*D contains a C-to-T transition at nucleotide 341 (codon 113), resulting in an Ala114-Val114 (A114V) substitution. GSTP1*C contains both these transitions [6], [7]. Enzymes with the valine at amino-acid 105 have a sevenfold higher catalytic efficiency for the diol epoxides of polycyclic aromatic hydrocarbons (PAH) than the isoenzymes with the isoleucine at this position. In contrast, the Val105 enzyme is threefold less effective with 1-chloro-2,4-dinitrobenzene as a substrate [6], [8], [9]. There is still no evidence of a functional effect of the A114V substitution alone (GSTP1*D), although it has been suggested that it augments the increased PAH activity of the I105V substitution (GSTP1*C) [8]. The missense substitution Ile105Val results from an A/G base substitution at nucleotide 313. The Val105 form of the *GSTP1* enzyme may be 2–3 times less stable than the canonical Ile105 form [10] and may be associated with a higher level of DNA adducts [11].

The association between *GSTP1* polymorphism and risk of HNC has been investigated, but these studies yielded controversial results. Some suggested that genetic polymorphisms of *GSTP1* genes could influence the balance between metabolic activation and detoxification of carcinogens and are therefore, related to individual susceptibility to HNC [12]–[16], other reports, however, did not support these findings [17]–[21]. Whether *GSTP1* polymorphism modifies the risk of HNC remains uncertain.

Meta-analyses have been conducted on the association between HNC and polymorphisms of *GSTM1* and *GSTT1* [22]–[24]. Additionally, a meta-analysis review of the association between HNC and *GSTM1*, *GSTT*, and *GSTP1* that included journal articles published between 1993 and 2003 was reported [25]. However, that paper included only a limited number of published studies on *GSTP1*, and results from new studies have been reported recently. Therefore, we performed the current meta-analysis, including journal articles published from 1997 to 2011, to more comprehensively investigate the association between *GSTP1* Ile05Val1 polymorphism and the risk of HNC.

## Materials and Methods

### Identification of Eligible Studies

To identify all articles that examined the association between the *GSTP1* Ile105Val polymorphism and risk of HNC, we conducted a literature search of PubMed using the following combination of keywords: Glutathione S-transferases P, polymorphism, and head and neck cancer, oral cancer/neoplasms, laryngeal cancer/neoplasms, pharyngeal cancer/neoplasms, or upper aerodigestive tract cancer/neoplasms. The language of publication was restricted to English.

### Inclusion and Exclusion Criteria

The following inclusion criteria were used for the literature selection: (a) case–control study methodology; (b) association of HNCs (including oral cancer, laryngeal cancer, pharyngeal cancer, and upper aerodigestive cancer) with *GSTP1* polymorphisms explored; (c) study sample size, odds ratios (ORs), and 95% confidence intervals (CIs) stated in the article; and (d) HNC cases confirmed using histopathology.

Major exclusion criteria were as follows: (a) aim and design of the study obviously different from our research objectives; (b) not case-control study; (c) control population included malignant tumor cases; and (d) article was a review or duplication of previous publication.

After performing the literature search, we reviewed all papers in accordance with the criteria defined above. In addition, the Hardy–Weinberg equilibrium (HWE) test was conducted to evaluate the genetic equilibrium of each study [26].

### Data Extraction

Two investigators (Lang and Song) reviewed and extracted information independently from selected publications in accordance with the criteria for inclusion and exclusion. Data were then entered into a database. Any conflicts over study/data inclusion were settled by a discussion between the investigators.

### Statistical Analysis

The crude odds ratios (ORs) and 95% confidence intervals (95% CIs) of *GSTP1* Ile105Val polymorphism and risk of HNC were estimated for each study. For detection of any possible sample size biases, the OR and its 95% CI to each study were plotted respectively against the number of participants. A Chi-square based I^2^-statistic test was performed to assess the potential heterogeneity among the studies. An I^2^ value of less than 25% indicates low heterogeneity, 25% to 50% indicates moderate heterogeneity, and greater than 50% indicates high heterogeneity. If the result of the heterogeneity test was p>0.05, ORs were pooled according to the fixed-effect model [27]. Otherwise, the random-effect model was used [28]. The significance of the pooled ORs was determined by the Z-test. The HWE was assessed via Fisher’s exact test. Publication bias was assessed by visual inspection of Begg’s funnel plots and linear regression, respectively [29], [30]. All statistical analyses were undertaken using the Stata 10.0 software program (Stata Corporation, College station, TX).

## Results

### Literature Search and Studies Characteristics

**Figure 1:**
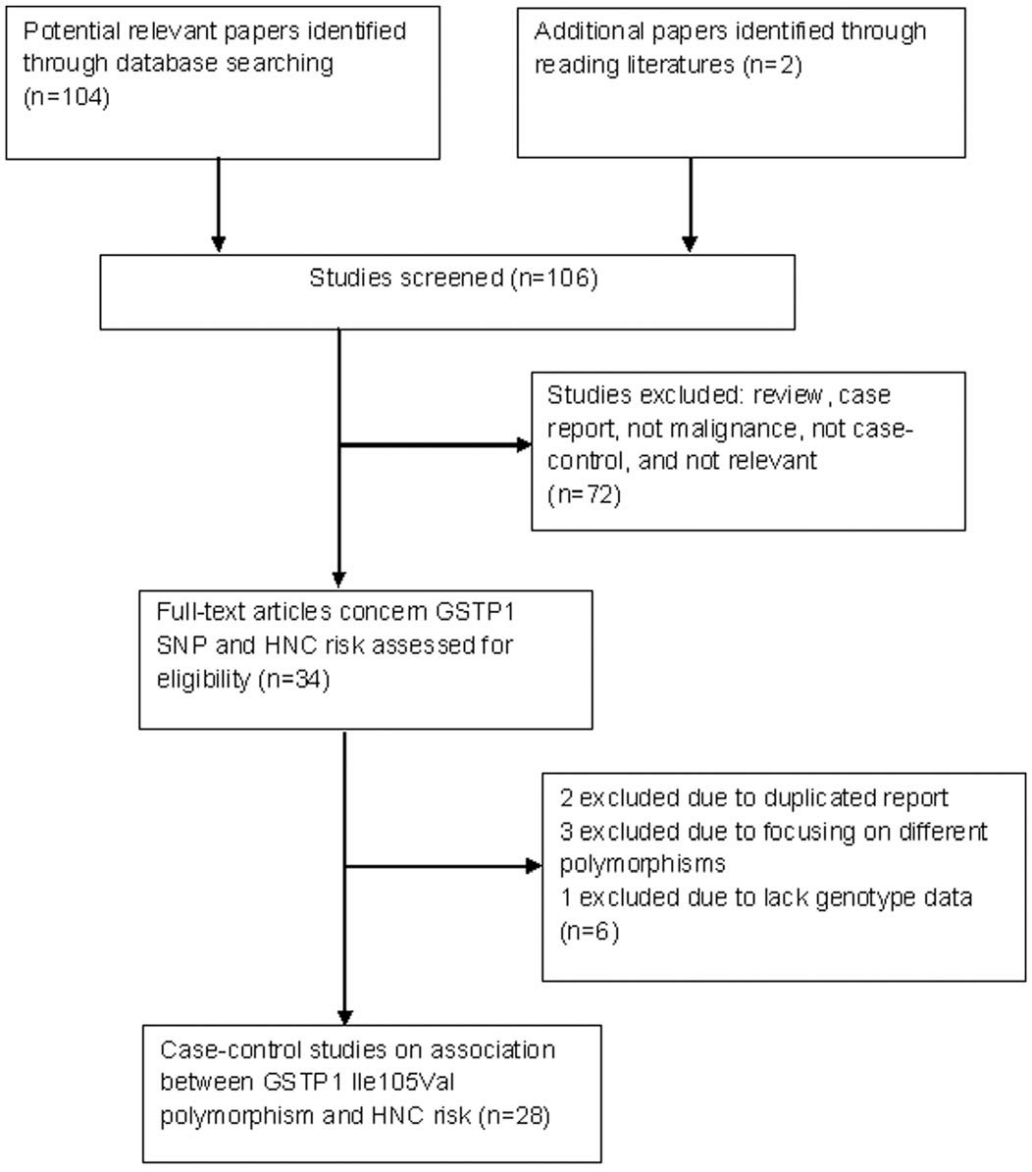
The flow diagram of included/excluded studies.

Our keyword search identified 104 papers and two additional relevant papers were adopted through reading literatures. Among them, 72 papers did not meet our criteria and were excluded after review of the abstracts. After reading the full texts of the remaining 34 papers, we eliminated an additional 6 papers, including 2 duplicated reports, 3 investigating different polymorphisms, and 1 lack of genotype data (Fig. 1). Therefore, a total of 28 case-control studies were identified, with 6404 cases and 6523 controls, of which 3136 cases and 3171 controls had the combined variant genotypes (Ile/Val and Val/Val) [12]–[21], [31]–[48]. The frequency of the *GSTP1* valine genotype was 23.8–72.7% among controls and 24.9–72.3% among cases. Among these 28 studies, 11 studies were performed in Asian populations, 11 in Caucasians, and 4 in “Whites”, in 2 studies the population was unclear. Controls in 6 studies were population-based and the other 22 studies adopted hospital-based population as controls. Nine papers focused on the oral cavity or oropharyngeal cancer, 2 papers on laryngeal cancer, 1 paper on nasopharyngeal cancer, and the other 16 papers on unspecific HNCs (including 2 papers that also provided data on non-HNCs). The study characteristics are listed in Table 1.

**Table 1:** Characteristics of eligible studies.

Author	Year	Country	Ethnicity	Genotyping Method	Tumor Site	Source of Control	Sample Size (case/control)	Matching		HWE	
Ruwali M	2011	India	Asian	PCR-RFLP	Head & Neck	HB	500/500		Age/Gender/Race		Y
Karen-Ng LP	2011	Malaysia	Asian	PCR-RFLP	Oral Cavity	HB	115/116		None		UK
Chen MK	2010	China	Asian	PCR-RFLP	Oral Cavity	HB	164/274		Age/Gender/Race		Y
Ruwali M	2009	India	Asian^a^	PCR	Head & Neck	HB	350/350		Age/Gender/Race		Y
Singh M	2008	India	Asian^a^	PCR	Head & Neck	HB	175/200		Age/Gender		N
Datta S	2007	India	Asian	PCR-RFLP	Oral Cavity	HB	307/209		Smoke		Y
Cho CG	2006	Korea	Asian	PCR	Head & Neck	HB	294/333		UK		Y
Cheng YJ	2003	China	Asian	PCR-RFLP	Nasopharynx	PB	264/323		UK		Y
Katoh T	1999	Japan	Asian	PCR-RFLP	Oral Cavity	HB	83/122		UK		N
Morita S	1999	Japan	Asian	PCR-RFLP	Head & Neck	PB	145/164		None		Y
Soya SS	2007	India	Asian	PCR-RFLP	Head & Neck	HB	408/222		Age/Gender/Race		Y
Oude Ophuis MB	2003	Netherland	Caucasian	PCR-RFLP	Head & Neck	HB	235/285		Age		Y
Kelders WP	2002	Netherland	UK^b^	PCR-RFLP	Head & Neck	HB	85/51		UK		Y
McWilliams JE	2000	USA	Caucasian	PCR-RFLP	Head & Neck	HB	146/124		Race/Alcohol		Y
Park JY	1999	USA	Caucasian + American	PCR-RFLP	Oral Cavity	HB	157/260		UK		N
Jourenkova-MN	1999	Europe	Caucasian	PCR-RFLP	Oral Cavity	HB	121/172		Age/Gender/Race/Smoke		Y
Jourenkova-MN	1999	France	Caucasian	PCR-RFLP	Larynx	HB	129/172		UK		Y
Matthias C	1998	Germany	Caucasian	PCR-RFLP	Head & Neck	HB	380/180		Race		N
To-Figueras J	2002	Spain	Caucasian	PCR-RFLP	Larynx	HB	204/203		None		Y
Soucek P	2010	Czech and Poland	Caucasian	PCR-RFLP	Head & Neck	PB	116/122		Age/Gender/Alcohol		Y
Reszka E	2008	Poland	Caucasian	PCR-RFLP	Head & Neck	PB	127/151		Gender/Alcohol/Occupation		UK
Harth V	2008	Germany	Caucasian	RT-PCR	Head & Neck	HB	312/300		UK		Y
Evans AJ	2004	USA	Caucasian	PCR-RFLP	Head & Neck	PB	283/208		None		Y
Leichsenring A	2006	Brazil	UK^b^	PCR-RFLP	Oral Cavity	HB	72/60		Gender		Y
Hataqima A	2008	Brazil	White+Black	PCR-RFLP	Oral Cavity	HB	231/212		Age/Gender/Race		Y
Peters ES	2006	USA	White^c^	PCR-RFLP	Head & Neck	PB	690/748		Age/Gender		Y
Amador AG	2002	USA	White^c^	PCR-RFLP	Head & Neck	HB	137/99		UK		N
Olshan AF	2006	USA	White^c^	PCR-RFLP	Head & Neck	HB	172/193		UK		Y

a male only;

b Brazilian;

c majority is White;

HWE, Hardy–Weinberg equilibrium; PCR-RFLP, polymerase chain reaction–restriction fragment length polymorphism; HB: Hospital based; PB: Population based; UK: Unknown or Unstated; Smoke: Tobacco consumption; Alcohol: Alcohol consumption. RT-PCR, Real-time–polymerase chain reaction.

### Test of Heterogeneity

**Figure 2:**
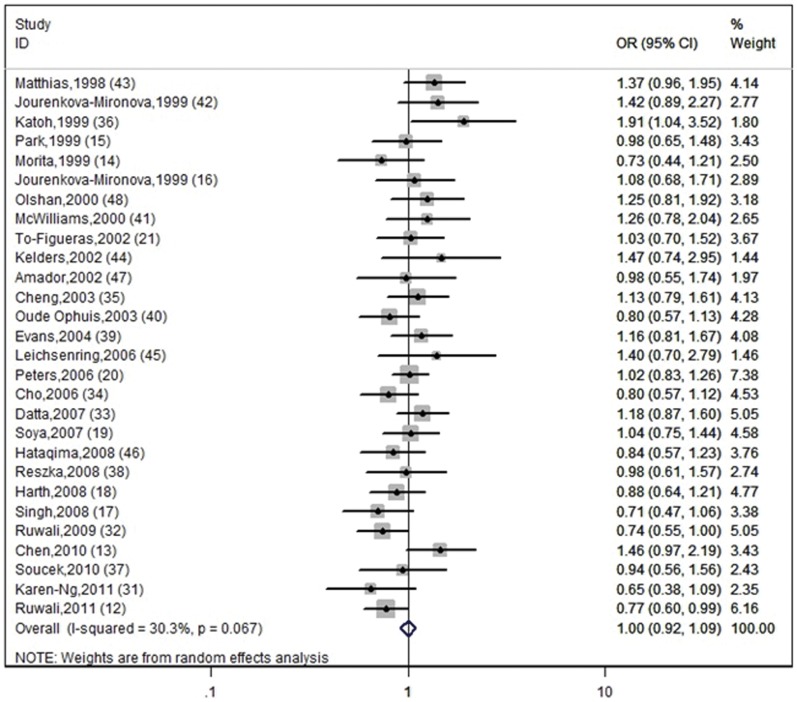
Forest plot with a random-effect model for GSTP1 Ile105Val polymorphism and risk for HNC. The center of each square represents the OR value, the area of each square is proportional to the sample size and thus the weight of the corresponding study, and the horizontal short line indicates the 95% confidence interval. The pooled OR is represented by the diamond. (Test for heterogeneity: chi^2^ = 38.75, df = 27, p = 0.067. Test for overall effect: z = 0.02, p = 0.984).

Figure 2 shows the association between the *GSTP1* Ile105Val polymorphism and risk of HNC. We analyzed the heterogeneity for all 28 studies and the test value of Chi-square was 38.62, with 27 degrees of freedom (d.f.) and 0.05<P<0.1 (p = 0.069). This result shows there is heterogeneity among the studies. Additionally, I^2^ value is calculated as another index for the heterogeneity test. As shown in Figure 2, the I^2^ value was 30.1% (between 25% to 50%), which suggests slight to moderate heterogeneity. Thus, the random-effect model was utilized for evaluation. In Figure 2, one can observe that 5 studies [13], [31], [36], [44], [45] may attribute to the major sources of heterogeneity. Further stratified meta-analysis is needed to perform.

### Meta-analysis Results

The summary OR for the *GSTP1* Ile105Val genotype was 1.00 (OR = 1.00, 95% CI = 0.92–1.09) and the test for overall effect Z value was 0.02 (p = 0.984). The overall meta-analysis showed that there was no significant association between the risk of HNC and *GSTP1* Ile105Val polymorphism (p>0.05). Figure 2 shows the pooled OR with 95% CI of association between the *GSTP1* Ile105Val polymorphism and risk of HNC.

To determine the cause of the moderate heterogeneity among the studies and to obtain more accurate results, we conducted further meta-analyses stratified according to tumor site, study sample size, ethnic group, publication year, source of controls, and consistency of frequency with HWE. In four unspecific “Head and Neck” tumor site studies, the sample size of subtype cancers were also available: thus, a total of 12 studies on oral and oropharyngeal cancers, 6 studies on laryngeal cancer, 1 on nasopharyngeal cancer, and 13 on mixed HNCs were examined in a stratified meta-analysis (Table 2). Meta-regression was employed to calculate the between-study variance. Publication year was identified as the main cause of heterogeneity. Only 8.22% of residual variation heterogeneity was left if we excluded the study year from the meta-analysis, and the estimate of between-study variance was tau = 0.000561, p = 0.005. The pooled OR of studies published before 2005 appeared to indicate an association between the *GSTP1* Ile105Val polymorphism and risk of HNC, although p>0.05. Meta-analyses stratified according to other factors, such as tumor site, source of controls, ethnicity, sample size, and consistency of HWE, did not show a significant association between the *GSTP1* Ile105Val polymorphism and risk of HNC (Table 2). The residual variation I^2^ values (heterogeneities) of stratified meta-regression were 26.1% of tumor site, 28.4% of ethnicity, 32.9% of source of control, 31.3% of sample size, 32.8% of HWE consistency respectively. Compared with the overall I^2^ value of 30.1%, none of these factors predominantly contribute to the overall heterogeneity, except publication year.

**Table 2:** Stratified Meta-analysis and Meta-regression Analysis of Heterogeneity.

Stratification	N	Meta-regression*	OR	95% CI	P value
Overall	28	I2=30.3%	1.00	0.921.10	0.984
Tumor site		P=0.157 (I2 res=26.1%)			
Mixed HNC	13		0.94	0.851.05	0.267
Oral/Oropharynx	12		1.10	0.901.33	0.355
Larynx	6		0.87	0.691.11	0.260
Nasopharynx	1		1.13	0.791.61	0.742
Ethnicity		P=0.208 (I2 res=28.4%)			
Asian	11		0.94	0.791.11	0.451
Caucasian	11		1.05	0.931.18	0.441
Other	6		1.05	0.901.22	0.549
Source of control		P=0.894 (I2 res=32.9%)			
Hospital-based	22		1.00	0.901.12	0.941
Population-based	6		1.02	0.891.18	0.762
Publication year		P=0.005 (I2 res=8.2%)			
Before 2005	14		1.12	0.991.26	0.066
2005 and after	14		0.92	0.821.03	0.142
Sample Size		P=0.363 (I2 res=31.3%)			
300	21		1.02	0.911.14	0.714
300	7		0.97	0.831.13	0.686
HWE		P=0.974 (I2 res=32.8%)			
Yes	21		1.00	0.911.09	0.933
No	7		1.01	0.781.30	0.972

* Meta-regression indicates the between-study variance.

** Includes studies without enough data to calculate HWE.

N, number; OR, odds ratio; 95%CI, confidence interval; “I^2^” indicates variation in OR attributable to heterogeneity; “I^2^ res” in the parentheses indicates residual variation due to heterogeneity of each factor; HWE, Hardy–Weinberg equilibrium.

### Sensitivity Analysis

In order to compare the differences between the meta-analyses and evaluate their sensitivity, we also reported the results of the fixed-effect model for *GSTP1*, as follows: the combined OR was 0.99 with 95% CI from 0.92 to 1.06 (z = 0.27, p = 0.790), similar to the results of the random-effect models (test of heterogeneity χ^2^ = 38.75, df = 27, p = 0.067).

### Bias Diagnostics

**Figure 3:**
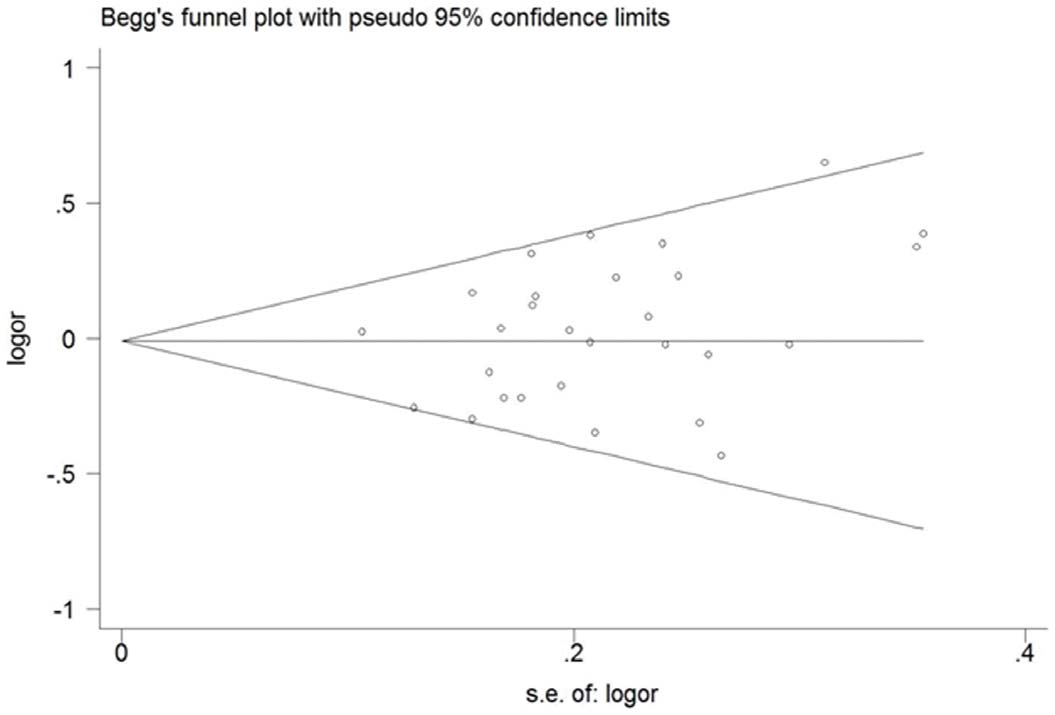
Begg’s funnel plot for publication bias assessment. Each hollow cycle represents a separate study for the indicated association.

A Begg’s funnel plot created to assess possible publication biases showed nearly symmetrical pattern, indicating that there was no publication bias (Fig. 3). In addition, Egger’s test used to quantitatively evaluate the publication bias, found no evidence of bias (p = 0.128).

## Discussion

*GSTP1* polymorphisms have been evaluated as risk factors for cancers in a number of studies. Extensive molecular epidemiological studies indicate that the *GSTP1* variant is more likely to lead to development of cancer than its wild type. A series of studies demonstrated that the *GSTP1* codon 105 polymorphism is associated with various types of cancer, including breast, prostate, and lung cancer [49]–[51]. However, in this meta-analysis of 28 case–control studies, there was no evidence supporting the hypothesis that the *GSTP1* Ile105Val polymorphism is significantly associated with risk of HNC in the general population.

One possible explanation for this lack of association may be suboptimal study design. Considering the role of *GSTP1* as a carcinogen-metabolizing gene, potential effect of tobacco and alcohol to HNC should be taken into consideration of the study design. As shown in Table 1, the rate of matched tobacco or alcohol consumption between case and control was low. There were only 2 studies [33], [42] with smoking matching and 3 with alcohol consumption matching [37], [38], [41]. Although adjustment according to smoking and alcohol has been done in most studies, this may still cause inevitable heterogeneity among studies.

There is also evidence of heterogeneity on other aspects among the studies in this systematic review and meta-analysis. Potential sources of heterogeneity include the publication year, case-control matching, and sample size. The pooled subgroup analysis of a subset of studies published before 2005 suggested a weak association, although it was not statistically significant (p = 0.066). The reason for this is not clear. It might be due to uncontrolled confounding factors or to inherent bias in the study design. It is clear from this meta-analysis that the design of some of the case–control studies was suboptimal. From the forest plot (Fig. 2), one can observe that 5 studies are the major sources of heterogeneity [13], [31], [36], [44], [45]. In some papers, the study design included important oversights, for example, some studies used small sample sizes [36], [44], [45]. Selection bias may be another source of heterogeneity. Some studies used samples with highly heterogeneous ethnic origins [45], [47] or the composition of ethnicity was not clearly stated [44]. Other studies recruited control subjects from hospital-based population. Since it is conceivable that the *GSTP1* gene might confer susceptibility to non-cancer disease, the genotype frequencies might be different between population-based and hospital-based controls, and this might introduce heterogeneity among studies. The use of population-based controls is, therefore, more appropriate in association studies.

Due to the fact that deviation from HWE may point to methodological weaknesses, such as biased selection of subjects, genotyping errors, or population stratification, we performed further stratified analyses. The meta-analyses that excluded studies whose genotype frequencies in controls significantly departed from HWE did not result in any substantial modification of crude OR results pertaining to the *GSTP1* Ile105Val (Table 2). Although studies with heterogeneity do not significantly alter the estimate of the overall OR and result in a type I error, more optimal and well-designed studies are needed to investigate this association more closely and systematically.

Studies from Asian countries tended to support the association between the *GSTP1* Ile105Val polymorphism and risk of HNC, whereas most studies from European countries failed to demonstrate this association. According to our results, after subgroup analysis by ethnicity, cancer sites, and source of control group, no significant associations were observed. However, ethnicity is definitely an important factor when investigating the association of genetic polymorphisms with cancer risk. Further large-scale investigation might be needed to validate our results.

Our results showed no association between the *GSTP1* Ile105Val polymorphism and the risk of HNC in general, as well as between this polymorphism and the risk of oral cancer or laryngeal cancer when we stratified HNC according to subtypes of tumor sites. Our results are consistent with those of Hashibe and colleagues, except the finding of the higher risk of oral cancer than the risk of laryngeal cancer for the *GSTP1* any valine genotype [25]. It was reported that the metabolic action of GST enzymes may differ by cancer site; the highest concentrations of GSTP1 have been observed in oral and pharyngeal tissues, and the highest concentrations of GSTM1 have been observed in laryngeal tissue, relative to the other GSTs [52]. Studies on *GSTP1* polymorphism and the risk of oral cavity cancer reached controversial conclusions [13], [15], [31], [36], [45]. In this study, no positive association was found between the *GSTP1* polymorphism and the risk of oral or oropharyngeal cancer. Since the data on subsets of oral cavity cancer and oropharyngeal cancer were not available, further stratified meta-analysis was not able to be performed. If there is association between the *GSTP1* Ile105Val polymorphism and the risk of oral cavity cancer or oropharyngeal cancer still remains unclear, although it is negative as combined subset according to our results.

Although considerable effort was made to test for the possible association between the *GSTP1* Ile105Val polymorphism and risk of HNC, there are still some limitations inherited from the published studies. First, due to limited detailed data presented in the published studies, the potential effect of important risk factors to HNC was not examined, such as smoking (data of sample size associated with smoking was available in only 7 studies) and alcohol consumption. Second, the results are only based on single-factor estimates, without adjustment for other risk factors such as age, ethnicity, family history, and environmental factors. Third, *GSTP1* may influence susceptibility to head and neck cancer independently or with other genes. However, due to lack of individual data in the present review, we did not perform more detailed analyses, such as analyses of joint effects with other risk factors or gene-gene or gene-environment interactions.

In conclusion, this meta-analysis demonstrates that the *GSTP1* Ile105Val polymorphism appears to not be associated with risk of HNC. To confirm our findings, well-designed studies with large sample sizes in diverse ethnic populations are warranted.

## Supporting Information

Figure S1 **Flowchart for selection of studies.**
(PDF)

Text S1 **PRISMA Checklist.**
(PDF)
