# Supplementary figures and images for: Association of GSTP1 Ile105Val Polymorphism and Risk of Head and Neck Cancers: A Meta-Analysis of 28 Case-Control Studies
Source: PLoS One. 2012 Nov 7;7(11):e48132. doi: 10.1371/journal.pone.0048132 (PMC3492338; doi:10.1371/journal.pone.0048132)

The flow diagram of included/excluded studies

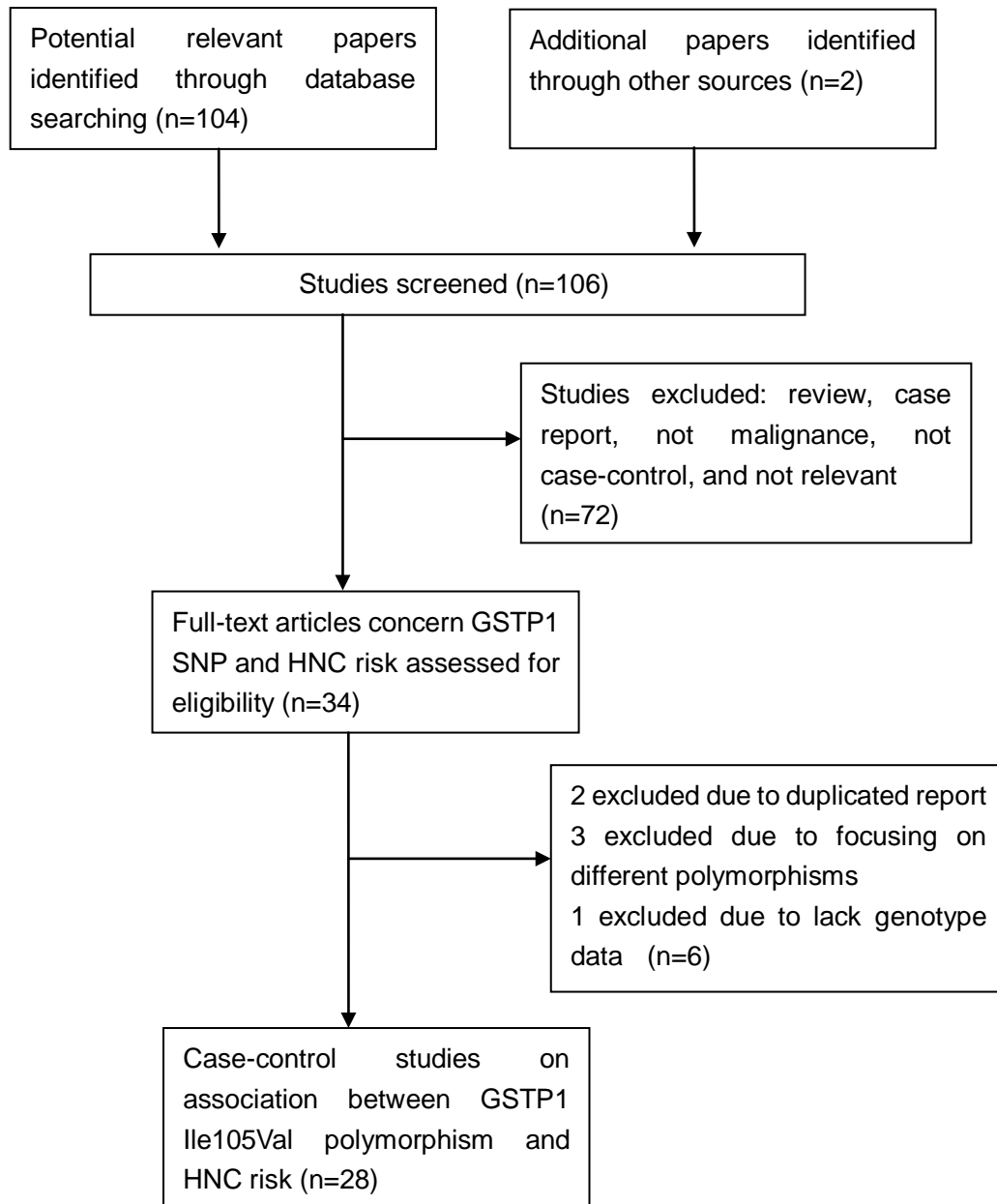

Supplement: Figure S1 — Flowchart for selection of studies. (PDF) [file pone.0048132.s001.pdf]
