# Supplementary material for: Association of GSTP1 Ile105Val Polymorphism and Risk of Head and Neck Cancers: A Meta-Analysis of 28 Case-Control Studies
Source: PLoS One. 2012 Nov 7;7(11):e48132. doi: 10.1371/journal.pone.0048132 (PMC3492338; doi:10.1371/journal.pone.0048132)
Supplement: Text S1 — PRISMA Checklist. (PDF) [file pone.0048132.s002.pdf]

PRISMA Checklist

for Meta-analysis on Association of *GSTP1* Ile105Val polymorphism and risk of head and neck cancers

Section/topic	#	Checklist item	Reported on page #
**TITLE**			
Title	1	Meta-analysis on Association of *GSTP1* Ile105Val polymorphism and risk of head and neck cancers	1
**ABSTRACT**			
Structured summary	2	**Background:** The *GSTP1* polymorphism have been considered a risk modifier for developing head and neck cancer (HNC) in many studies; however, the results of such studies are inconsistent;<br>**Objectives:** To evaluate the possible association between the *GSTP1* Ile105Val polymorphism and risk of HNC;<br>**Data sources:** A literature search of PubMed has been conducted and 28 case--control studies were included finally;<br>**Study eligibility criteria:** (a) case–control study methodology; (b) association of HNCs (including oral cancer, laryngeal cancer, pharyngeal cancer, and upper aerodigestive cancer) with *GSTP1* polymorphisms explored; (c) study sample size, odds ratios (ORs), and 95% confidence intervals (CIs) stated in the article; and (d) HNC cases confirmed using histopathology;<br>**Participants and interventions**: Two investigators (Lang and Song) reviewed and extracted information independently, and any conflicts over study/data inclusion were settled by a discussion between the investigators;<br>**Study appraisal and synthesis methods:** The crude odds ratios (ORs) and 95% confidence intervals (95% CIs) of *GSTP1* Ile105Val polymorphism and risk of HNC were estimated for each study. A Chi-square—based I^2 test was performed to assess the potential heterogeneity among the studies. Pooled OR with 95% CI were assessed using both fixed-effects and random-effects models. The	2

Section/topic	#	Checklist item	Reported on page #
		significance of the pooled OR was determined by the Z-test.;<br>**Results:** The *GSTP1* Ile105Val polymorphism was not significantly associated with risk of HNC in the overall study population (pooled OR 1.0, 95% CI 0.9–1.1) or in subgroup analyses stratified by ethnicity, sample size, tumor site or publication year;<br>**Limitations:** This meta-analysis is only based on single-factor estimates, without adjustment for other risk factors. Due to lack of individual data in the present review, more detailed analyses, such as analyses of joint effects with other risk factors or gene-gene or gene-environment interactions , has not been performed;<br>**Conclusions:** This meta-analysis demonstrates that the *GSTP1* Ile105Val polymorphism appears to not be associated with risk of HNC.	
**INTRODUCTION**			
Rationale	3	Recent evidence indicates that polymorphisms in carcinogen-metabolizing genes play critical roles in determining individual susceptibility to HNC. One of the them is *GSTP1* Ile105Val polymorphism. However, the results of previous studies are inconsistent. Whether *GSTP1* polymorphism modifies the risk of HNC remains uncertain and needs further study with large sample sizes. A meta-analysis review on this issue was reported but the studies included in that paper was limited and lack of newly reported data since 2003. Therefore we performed the current meta-analysis, including journal articles published from 1997 to 2011. Our analysis aims to minimize the issue of the selection bias, and, therefore, greatly improves the accuracy of the association in this analysis.	4
Objectives	4	The following issues have been discussed and addressed:<br>1. This meta-analysis focuses on the association of *GSTP1* Ile105Val polymorphism and risk of head and neck cancers. Other SNP loci and thyroid cancer or esophageal cancer related studies should be excluded;<br>2. Searching keywords selection:” cancer” or “neoplasms” should be used;	4

Section/topic	#	Checklist item	Reported on page #
		3. After primary searching , Dr. Lang and Dr. Song are responsible to review and assess the studies independently. Different opinion will be promoted and discussed by conference of all investigators.<br>4. Stratified meta-analyses may need to be done according to publication year, tumor site, sample size, source of controls, ethnicity, and consistency of HWE. While variables of country, genotype methods and matching criteria will not be considered because of less affect.	
**METHODS**			
Protocol and registration	5	Review protocol was completed before the review started. However, it cannot be accessed on line and we have no registration number.	
Eligibility criteria	6	The following inclusion criteria were used for the literature selection: (a) case–control study methodology; (b) association of HNCs (including oral cancer, laryngeal cancer, pharyngeal cancer, and upper aerodigestive cancer) with *GSTP1* polymorphisms explored; (c) study sample size, odds ratios (ORs), and 95% confidence intervals (CIs) stated in the article; and (d) HNC cases confirmed using histopathology. No limitation fro published year, and language of articles was restricted to English.	5
Information sources	7	database (Pubmed) searching coverage:1/1/1980-- 4/4/2012 .<br>Date of last searched: 2012,April.	5

Section/topic	#	Checklist item	Reported on page #
Search	8	Database: PubMed.<br>Search steps:<br>a. Search by key word "Glutathione S-transferases P";<br>b. Search by key word "polymorphism";<br>c. Search "a" AND "b";<br>d. Search "head neck cancer";<br>e. Search "oral cancer" or " oral neoplasms";<br>f. Search "pharyngeal cancer" or "pharyngeal neoplasms";<br>g. Search "laryngeal cancer" or "laryngeal neoplasms";<br>h. Search "upper aerodigestive tract cancer" or "upper aerodigestive tract neoplasms";<br>i. Search "d" OR "e" OR "f" OR "g" OR "h";<br>j. Search "c" AND "i"	5
Study selection	9	Our keyword search identified 104 papers and two additional relevant papers were adopted through reading literatures. Two investigators (Lang and Song) reviewed and extracted information from the papers independently. Among them, 72 papers did not meet our criteria and were excluded after review of the abstracts. After reading the full texts of the remaining 34 papers, we eliminated an additional 6 papers, including 2 duplicated reports, 3 investigating different polymorphisms, and 1 lack of genotype data. The left 28 eligible case-control studies with 6404 cases and 6523 controls were evaluated regarding to the sample size, ethnicity, tumor site, experimental methods, control group selection and consistency with HWE.	6<br>Figure 1
Data collection process	10	Investigators reviewed the paper and extract data information into a excel form, according to the following variables (see section 11). Then the data will be checked and confirmed by investigators group, and issues of duplication or inconsistency will be addressed by discussion.	5

Section/topic	#	Checklist item	Reported on page #
Data items	11	Study number;<br>Author: name of the first author;<br>Publication year: time showing on the paper;<br>Country: which the corresponding institution belong to;<br>Ethnicity: Asian/Caucasion/ White/Black/African;<br>Control source: Hospital-based/ Population-based; Healthy volunteer/Patients with other disease/ Patients with cancer;<br>Cancer Category: Oral cavity Ca/Oropharyngeal Ca/Nasopharyngeal Ca/Laryngeal Ca/Mixed HNC;<br>Pathology confirmed: Yes/No;<br>Polymorphism Loci;<br>Genotyping methods: PCR-RFLP/RT-PCR/Other;<br>Matching criteria: Case-Control groups matching items, e.g. race/age/gender/smoking or alcohol consumption;<br>HWE consistency: genotype frequency in control group consistent with HWE or not;<br>Sample size: case number/SNP-in-Case number/control number/ SNP-in-Control number;<br>OR and 95%CI.	18
Risk of bias in individual studies	12	Bias in individual studies was identified through evaluation of the data items above such as sample size, ethnicity and HWE consistency. Studies with too small sample size (<50) will not be included into data synthesis. Mixed ethnicity composing of subjects will be excluded from subgroup analysis of ethnicity.	6
Summary measures	13	The ORs and 95% CIs of were estimated for each study. For detection of any possible sample size biases, the OR and its 95% CI to each study were plotted respectively against the number of participants. A Chi-square—based I^2-statistic test was performed to assess the potential heterogeneity among the studies.	6

Section/topic	#	Checklist item	Reported on page #
		If the result of the heterogeneity test was p > 0.05, ORs were pooled according to the fixed-effect model. Otherwise, the random-effect model was used. The significance of the pooled ORs was determined by the Z-test. The HWE was assessed via Fisher's exact test. Publication bias was assessed by visual inspection of Begg's funnel plots and linear regression, respectively. All statistical analyses were undertaken using the Stata 10.0 software program.	
Synthesis of results	14	Additionally, I-square value is calculated as another index for the heterogeneity test. An I^2 value of less than 25% indicates low heterogeneity, 25% to 50% indicates moderate heterogeneity, and greater than 50% indicates high heterogeneity. If the result of the heterogeneity test was p > 0.05, ORs were pooled according to the fixed-effect model. Otherwise, the random-effect model was used. The significance of the pooled ORs was determined by the Z-test.	6
Risk of bias across studies	15	Bias of studies first can be observed through their OR and 95% plotted in the Forest Plot. Begg's funnel plot and Egger's test were used to quantitatively evaluate the publication bias.	6
Additional analyses	16	Further meta-analyses stratified according to tumor site, study sample size, ethnic groups, publication years, source of controls, and consistency of frequency with HWE. Meta-regression was employed to calculate the between-study variance. The fixed-effect model meta-analysis was also conducted to compare the differences between the meta-analyses and evaluate their sensitivity.	6
**RESULTS**			
Study selection	17	Our keyword search identified 104 papers and two additional relevant papers were adopted through reading literatures. Among them, 72 papers did not meet our criteria and were excluded after review of the abstracts. After reading the full texts of the remaining 34 papers, we eliminated an additional 6 papers, including 2 duplicated reports, 3 investigating different polymorphisms, and 1	6,7

Section/topic	#	Checklist item	Reported on page #
		lack of genotype data(Fig.1). Therefore, a total of 28 case-control studies were identified, with 6404 cases and 6523 controls.	
Study characteristics	18	The following data were extracted and analyzed :<br>Author name, Publication year, Country, Ethnicity of participants, Control source, Cancer category, Pathology, Polymorphism Loci, Genotyping methods, Matching criteria, HWE consistency, Sample size, OR and 95%CI.<br>(Table 1)	18
Risk of bias within studies	19	Among these 28 studies, the ethinity of 4 studies employed mixed population and it was unclear in 2 studies[ref.44 and 45].<br>Controls in 6 studies were population-based and the other 22 studies adopted hospital-based population as controls.	7
Results of individual studies	20	The OR and 95% CI of each study were plotted respectively against the number of participants and shown in Figure 2.	Fig 2
Synthesis of results	21	Table 2 and Figure 2. Additional supporting information.	19
Risk of bias across studies	22	Five studies [13,31,36,44,45] may attribute to the major sources of heterogeneity. Further stratified meta-analysis were performed.	7
Additional analysis	23	Publication year was identified as the main cause of heterogeneity. However, meta-analyses stratified according to publication year, tumor site, sample size, source of controls, ethnicity, and consistency of HWE, did not show a significant association between the *GSTP1* Ile105Val polymorphism and risk of HNC.	8
**DISCUSSION**			
Summary of evidence	24	In this meta-analysis of 28 case—control studies, there was no evidence supporting the hypothesis that the *GSTP1* Ile105Val polymorphism is significantly associated with risk of HNC in the general population. To confirm our findings, well-designed studies with large sample sizes in diverse ethnic populations are warranted.	10
Limitations	25	The results are only based on single-factor estimates, without adjustment for	11

Section/topic	#	Checklist item	Reported on page #
		other risk factors such as age, ethnicity, family history, and environmental factors. Due to lack of individual data in the present review, we did not perform more detailed analyses, such as analyses of joint effects with other risk factors or gene-gene or gene-environment interactions.	
Conclusions	26	This meta-analysis demonstrates that the *GSTP1* Ile105Val polymorphism appears to not be associated with risk of HNC. Well-designed studies with large sample sizes in diverse ethnic populations are warranted.	11
**FUNDING**			
Funding	27	No	
